# Chitosan-Based Taurine Nanoparticles Alleviate Dexamethasone-Induced Pulmonary–Thyroid Axis Dysfunction via Redox–Inflammatory Signaling Modulation in Rats

**DOI:** 10.3390/ijms27094072

**Published:** 2026-05-01

**Authors:** Amany M. Hamed, Ahmed M. Refaat, Safaa S. Soliman, Dalia A. Elbahy, Rasha Abdeen Refaei, Alia K. H. Mahmoud, Ahmed S. Osman, Safaa Mohammed Elmahdy, Eman E. Ragab, Hany M. R. Abdel-Latif, Ahmed Mohamed Mahmoud Abdelfattah Elkassas, Ahmed R. H. Ahmed, Elsayed Eldeeb Mehana Hamouda, Azza M. A. Abouelella

**Affiliations:** 1 Chemistry Department, Faculty of Science, Sohag University, Sohag 82524, Egypt; 2 Department of Zoology, Faculty of Science, Minia University, Minia 61519, Egypt; 3 Department of Clinical Pharmacology, Faculty of Medicine, Sohag University, Sohag 82524, Egypt; 4 Department of Physiology, Faculty of Medicine, Sohag University, Sohag 82524, Egypt; 5 Department of Medical Biochemistry and Molecular Biology, Faculty of Medicine, Sohag University, Sohag 82524, Egypt; 6 Department of Biochemistry, Faculty of Veterinary Medicine, Sohag University, Sohag 82524, Egypt; 7 Department of Anatomy, Faculty of Medicine, Sohag University, Sohag 82524, Egypt; 8 Histology department, Faculty of Medicine, Sohag University, Sohag 82524, Egypt; 9 Department of Poultry and Fish Diseases, Faculty of Veterinary Medicine, Alexandria University, Alexandria 26571, Egypt; 10 College of Medicine, Alexandria University, Alexandria 26571, Egypt; 11 Department of Pathology, Faculty of Medicine, Sohag University, Sohag 82524, Egypt; 12 Department of Pathology, College of Veterinary Medicine, Alexandria University, Alexandria 26571, Egypt

**Keywords:** taurine nanoparticles, dexamethasone, oxidative stress, thyroid and lung histopathology, MPO

## Abstract

Dexamethasone induces systemic toxicity, including oxidative stress, inflammation, hematological disturbances, and organ damage, particularly in the lungs and thyroid. Taurine exhibits antioxidant and anti-inflammatory properties, but poor bioavailability limits its efficacy. Nanoparticle delivery may enhance stability and tissue targeting. This study aimed to evaluate the protective effects of taurine-loaded chitosan nanoparticles (Tau–CS NPs) against dexamethasone-induced tissue injury in rats. Forty-eight male Wistar rats were allocated into control, DEXA, DEXA + silymarin, DEXA + taurine, and DEXA + Tau–CS NPs groups. Tau–CS NPs were characterized by TEM, UV–vis, FTIR, encapsulation efficiency, and drug loading. Hematology, oxidative stress markers (CAT, SOD, GSH, MDA), thyroid hormones (T3, T4, TSH, calcitonin), protein profile, lung and thyroid histopathology, and MPO expression were assessed. Tau–CS NPs showed uniform spherical morphology (11–60 nm), high encapsulation (98.2%), and substantial loading (50.36%). Dexamethasone caused hematological, oxidative, thyroidal, and histological disturbances. Tau–CS NPs markedly restored hematological indices, antioxidant defenses, thyroid function, protein profile, and tissue architecture, outperforming free taurine and silymarin. MPO expression was significantly reduced, indicating decreased inflammation. Taurine nanoparticles effectively mitigate dexamethasone-induced systemic and organ-specific toxicity, offering improved bioavailability and targeted delivery, highlighting their therapeutic potential.

## 1. Introduction

Dexamethasone, a potent synthetic glucocorticoid widely used for its anti-inflammatory and immunosuppressive properties, is extensively prescribed in clinical settings for conditions ranging from autoimmune diseases to severe infections and respiratory distress syndromes [1]. Despite its therapeutic efficacy, chronic or high-dose dexamethasone administration has been associated with significant adverse effects, including oxidative stress, immune dysregulation, and tissue damage in multiple organs [2]. Although the lung is a primary target for inflammatory insults, glucocorticoid-induced pulmonary injury has been increasingly recognized, manifested by oxidative damage, inflammatory cell infiltration, and disruption of alveolar architecture. These pathological changes not only compromise respiratory function but also reflect the complex interplay between exogenous corticosteroid use and redox imbalance within pulmonary tissue. Such toxicity can further extend to other endocrine organs, including the thyroid gland, where disruption of hormone synthesis and feedback regulation has been documented in animal models following steroid exposure [3].

At the molecular level, oxidative stress and inflammatory responses are critical mediators of glucocorticoid-induced organ injury. Excess reactive oxygen species (ROS) production and lipid peroxidation have been implicated in membrane damage, enzyme inactivation, and DNA strand breaks, ultimately leading to cell death and tissue dysfunction. This imbalance in redox homeostasis is often accompanied by dysregulation of endogenous antioxidant defenses such as superoxide dismutase (SOD), catalase (CAT), and reduced glutathione (GSH). In parallel, pro-inflammatory signaling pathways, including p38/MAPK and NF-κB, drive the synthesis of cytokines and chemokines, exacerbating local inflammatory responses [4]. Consequently, there is a critical need for interventions capable of attenuating both oxidative and inflammatory cascades triggered by dexamethasone toxicity.

Chronic dexamethasone exposure has been increasingly associated with multisystem toxicity extending beyond its classical immunosuppressive effects, particularly through disruption of endocrine and pulmonary homeostasis. Excess glucocorticoid administration can suppress the hypothalamic–pituitary–thyroid (HPT) axis by inhibiting thyrotropin-releasing hormone (TRH) and thyroid-stimulating hormone (TSH) secretion, ultimately reducing circulating thyroid hormones and impairing thyroid gland function. In addition, dexamethasone-induced oxidative stress may directly damage thyroid follicular cells through excessive reactive oxygen species (ROS) generation, lipid peroxidation, and mitochondrial dysfunction. Pulmonary tissues are similarly vulnerable, as prolonged dexamethasone exposure may provoke redox imbalance, inflammatory cytokine dysregulation, alveolar structural damage, and impaired respiratory defense mechanisms. These combined pulmonary–thyroid alterations highlight the importance of investigating protective therapeutic strategies capable of modulating oxidative and inflammatory pathways during glucocorticoid-induced systemic toxicity [5,6].

Taurine, a naturally occurring sulfur-containing amino acid, has emerged as a promising cytoprotective molecule due to its potent antioxidant and anti-inflammatory properties. Taurine not only directly scavenges ROS but also enhances antioxidant defense systems, stabilizes cellular membranes, and modulates calcium homeostasis, thereby preserving cellular integrity under stress conditions [7,8]. A growing body of evidence supports taurine’s protective effects in diverse models of tissue injury: for instance, exogenous taurine supplementation has been shown to reduce lipid peroxidation, restore GSH levels, and suppress pro-inflammatory cytokine production in experimental lung injury [9].

Despite taurine’s well-documented antioxidant, anti-inflammatory, and cytoprotective properties, its broader clinical application may be constrained by pharmacokinetic limitations such as rapid systemic elimination, significant renal clearance, and limited tissue-specific retention following conventional administration. Human pharmacokinetic studies indicate that taurine possesses a relatively short plasma half-life, which may reduce sustained therapeutic concentrations in target organs and necessitate repeated dosing. Additionally, taurine uptake depends largely on transporter-mediated mechanisms, potentially restricting efficient accumulation under pathological conditions. To overcome these limitations, nanoparticle-based delivery systems have emerged as promising therapeutic platforms capable of enhancing bioavailability, protecting active compounds from premature degradation, prolonging systemic circulation, and enabling controlled or tissue-specific release. Chitosan nanoparticles, in particular, offer favorable biocompatibility, biodegradability, and mucoadhesive properties that may substantially improve taurine delivery efficiency. Therefore, taurine-loaded chitosan nanoparticles may represent an advanced therapeutic strategy to enhance taurine’s protective efficacy against dexamethasone-induced pulmonary and thyroid toxicity by improving pharmacokinetic performance and targeted biological action [10,11,12].

Given the scarcity of studies addressing the combined pulmonary and thyroid toxicity of dexamethasone and the limited data on nanoparticle-mediated taurine therapy, this study was designed to fill a critical gap in current preclinical research. Specifically, we investigated whether taurine nanoparticles can mitigate dexamethasone-induced pulmonary and thyroid dysfunction by modulating oxidative stress markers, inflammatory responses, and histopathological outcomes in a rat model. Elucidating these protective mechanisms is essential not only for understanding the systemic impact of glucocorticoid toxicity but also for advancing novel therapeutic strategies that leverage nanotechnology to enhance antioxidant and anti-inflammatory defenses.

## 2. Results

### 2.1. Taurine-Loaded Chitosan Nanoparticles Characterization

The physicochemical characterization of taurine-loaded chitosan nanoparticles (Tau–CS NPs) confirmed successful fabrication and effective taurine loading within the chitosan matrix, as evidenced by TEM, particle size distribution, UV–visible spectroscopy, and FTIR analysis.

**Figure 1:**
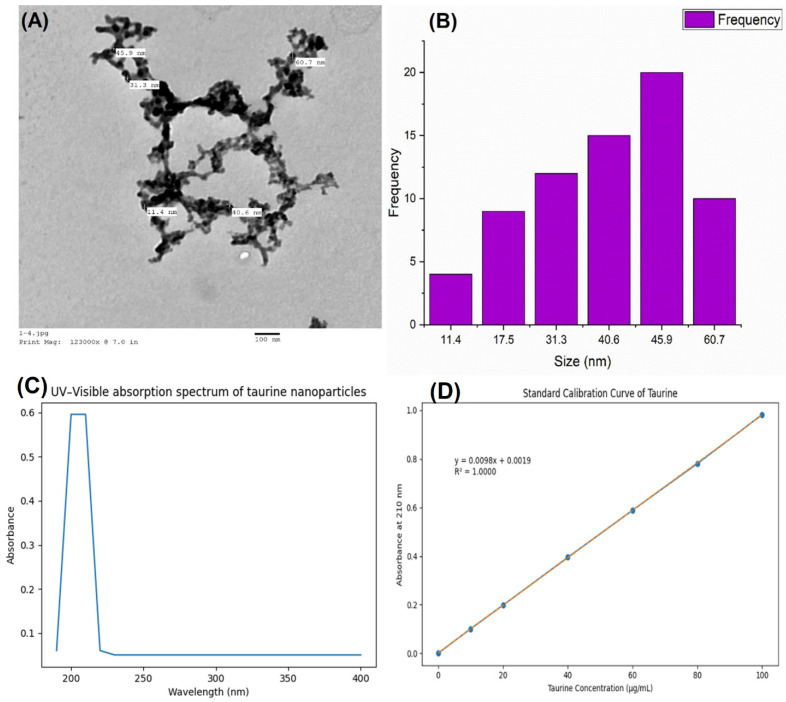
Characterization of taurine-loaded chitosan nanoparticles (Tau–CS NPs). (**A**) Transmission electron microscopy (TEM) micrograph showing the morphology and nanoscale size of Tau–CS NPs. The nanoparticles exhibit a predominantly spherical shape with good dispersion. (**B**) Particle size distribution histogram derived from TEM analysis, showing a narrow size range with particle diameters primarily between 11.4 and 60.7 nm. (**C**) UV–visible absorption spectrum of Tau–CS NPs, exhibiting a characteristic absorption band in the UV region, which confirms the successful incorporation of taurine into the chitosan nanostructure. (**D**) Standard calibration curve of taurine measured by UV–visible spectrophotometry at 210 nm for the determination of free taurine concentration during nanoparticle encapsulation efficiency analysis.

Transmission electron microscopy (TEM) analysis (Figure 1A) revealed that taurine-loaded chitosan nanoparticles exhibited a predominantly spherical to quasi-spherical morphology with good dispersion and limited aggregation, indicating effective nanoparticle formation. The particle size ranged approximately from 11.4 to 60.7 nm, confirming the nanoscale dimension suitable for enhanced biological interaction and cellular uptake. The smooth surface and uniform contrast further support the successful encapsulation of taurine within the chitosan framework.

The particle size distribution histogram (Figure 1B) demonstrated a relatively narrow size distribution, with the majority of nanoparticles falling within the 45.9–55.4 nm range, representing the highest frequency. This distribution pattern suggests good size homogeneity and controlled nanoparticle synthesis, which is a critical parameter for reproducible biological performance.

The UV–visible absorption spectrum (Figure 1C) of taurine-loaded chitosan nanoparticles exhibited a characteristic absorption band in the UV region around 200–220 nm, which is consistent with the electronic transitions associated with taurine functional groups. The appearance and stabilization of this absorption peak in the nanoparticle formulation, compared to free taurine, indicate successful incorporation of taurine into the chitosan nanostructure rather than simple physical mixing.

Taurine quantification was validated using a standard calibration curve (Figure 1D) at λmax = 210 nm over the concentration range of 0–100 µg/mL. The calibration demonstrated excellent linearity (y = 0.0098x + 0.0019, R^2^ = 1.000), confirming the reliability and reproducibility of the UV–visible spectrophotometric method for determining encapsulation efficiency.

**Figure 2:**
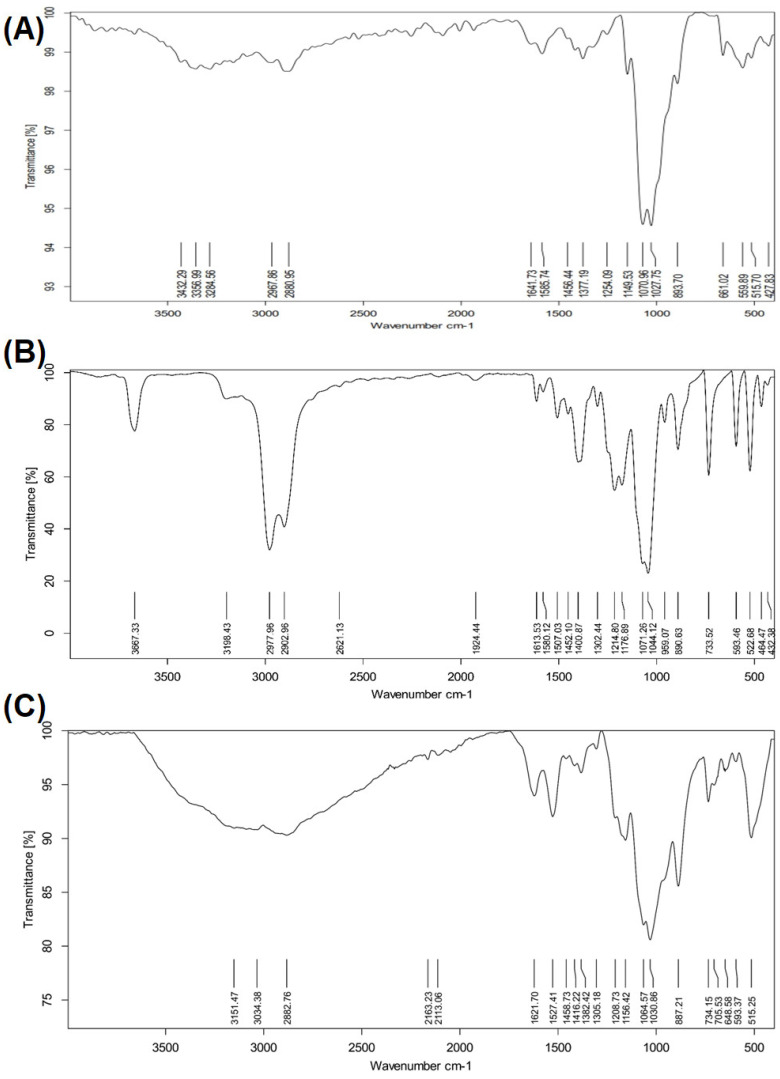
Fourier-transform infrared (FTIR) spectra of (**A**) chitosan, (**B**) free taurine, and (**C**) taurine-loaded chitosan nanoparticles (Tau-CS NPs). The spectra reveal characteristic functional groups of chitosan and taurine. Notable peak shifts, band broadening, and changes in intensity in the Tau-CS NP spectrum indicate strong intermolecular interactions between taurine and chitosan, confirming the successful encapsulation of taurine.

FTIR spectroscopy (Figure 2) provided further molecular-level confirmation of taurine loading. The FTIR spectrum of pure chitosan (Figure 2A) showed characteristic bands corresponding to O–H and N–H stretching vibrations around 3300–3500 cm^−1^, C–H stretching near 2900 cm^−1^, and amide I and II bands at approximately 1650 and 1550 cm^−1^. The spectrum of free taurine (Figure 2B) displayed distinct absorption peaks related to –NH_3_^+^ stretching, sulfonate (–SO_3_^−^) groups, and C–N vibrations, confirming its molecular fingerprint. In the taurine-loaded chitosan nanoparticles (Figure 2C), noticeable peak shifts, reduced intensities, and band broadening were observed, particularly in the regions associated with –NH, –OH, and sulfonate groups. These spectral changes indicate strong intermolecular interactions, such as hydrogen bonding and electrostatic interactions, between taurine and chitosan, confirming successful encapsulation rather than surface adsorption. These results demonstrate that taurine was efficiently loaded into chitosan nanoparticles, yielding stable nanostructures with uniform morphology, nanoscale size distribution, characteristic UV absorption behavior, and clear molecular interactions as evidenced by FTIR analysis.

#### Encapsulation Efficiency and Drug Loading Capacity of Taurine-Loaded Chitosan Nanoparticles

After the preparation of taurine-loaded chitosan nanoparticles, the nano-formulation was centrifuged to separate the nanoparticles from the unencapsulated taurine. The amount of free taurine in the supernatant was quantified using UV–visible spectrophotometry (UviLine 9400) at λmax ≈ 210 nm. Based on the spectrophotometric analysis, a small fraction of taurine (1.8%) remained unencapsulated. The encapsulation efficiency (EE%) was calculated to be 98.2%, indicating highly efficient incorporation of taurine within the chitosan matrix. Furthermore, the drug loading capacity (DLC%) was determined to be 50.36%, reflecting a high taurine content relative to the total nanoparticle mass.

### 2.2. Assessment of Hematological Parameters

As shown in Table 1, administration of dexamethasone (DEXA) induced marked hematological alterations compared to the control group. DEXA-treated rats showed a significant reduction in hemoglobin concentration (Hgb) accompanied by a pronounced increase in total leukocyte count, neutrophil percentage, and a significant decrease in lymphocyte, eosinophil, and monocyte percentages (*p* < 0.001), indicating a clear inflammatory and immunological imbalance.

Co-treatment with silymarin significantly improved all hematological parameters compared to the DEXA group (*p* < 0.01), although values remained significantly different from the control group. Similarly, taurine administration markedly ameliorated DEXA-induced hematological disturbances (*p* < 0.05 vs. DEXA), with more pronounced normalization of leukocyte differentials.

Notably, treatment with taurine-loaded nanoparticles (Tau-NPs) demonstrated the most effective protective effect, restoring hemoglobin levels and leukocyte indices to values comparable to the control group, with no significant differences observed vs. the control. These findings indicate that taurine nanoformulation provides superior hematological protection against dexamethasone-induced toxicity compared to free taurine and silymarin.

### 2.3. Oxidative Stress and Antioxidant Status in Lung Tissue

As shown in Table 2, dexamethasone administration induced severe oxidative stress in lung tissue, evidenced by a significant reduction in antioxidant enzyme activities, including catalase (CAT), superoxide dismutase (SOD), and reduced glutathione (GSH), concomitant with a marked elevation in lipid peroxidation marker malondialdehyde (MDA), compared with the control group (*p* < 0.001).

Co-treatment with silymarin significantly attenuated dexamethasone-induced oxidative damage, as evidenced by a partial restoration of antioxidant defenses and a substantial reduction in MDA levels compared to the DEXA group (*p* < 0.01). Similarly, taurine administration markedly improved lung antioxidant status, with a more pronounced increase in CAT, SOD, and GSH levels and a greater reduction in MDA content (*p* < 0.05–0.01 vs. DEXA).

Notably, treatment with taurine-loaded nanoparticles (Tau-NPs) exhibited the most potent antioxidant effect, effectively restoring CAT, SOD, and GSH levels to values comparable to the control group, with no significant differences observed. Moreover, lipid peroxidation was markedly suppressed, as reflected by near-normal MDA levels. These findings highlight the superior protective efficacy of taurine nanoformulation against dexamethasone-induced oxidative stress in lung tissue.

### 2.4. Thyroid Oxidative Stress, Inflammation, and Detoxification Status

As indicated in Table 3, dexamethasone administration induced marked oxidative and inflammatory damage in thyroid tissue, as evidenced by a significant elevation in malondialdehyde (MDA) and nitric oxide (NO) levels, accompanied by pronounced depletion of antioxidant defenses, including superoxide dismutase (SOD), reduced glutathione (GSH), and quinone reductase (QR), in comparison with the control group (*p* < 0.001). In addition, gamma-glutamyl transferase (GGT) activity was markedly increased, reflecting impaired thyroid detoxification capacity.

Co-treatment with silymarin partially mitigated dexamethasone-induced thyroid injury, as demonstrated by significant reductions in MDA, NO, and GGT levels, along with moderate restoration of antioxidant and detoxification markers (*p* < 0.01–0.001 vs. DEXA). Taurine treatment exerted a more pronounced protective effect, significantly improving oxidative balance and reducing inflammatory burden in thyroid tissue (*p* < 0.01 vs. DEXA).

Notably, taurine-loaded nanoparticles (Tau-NPs) showed the greatest efficacy, markedly restoring antioxidant and detoxification parameters toward normal levels. GSH content returned to values comparable to the control group (NS), while MDA, NO, QR, and GGT levels were significantly improved compared with the DEXA group. These findings indicate that taurine nanoformulation offers superior protection against dexamethasone-induced oxidative, inflammatory, and detoxification disturbances in thyroid tissue.

### 2.5. Thyroid Hormonal Profile and Calcitonin Levels

**Figure 3:**
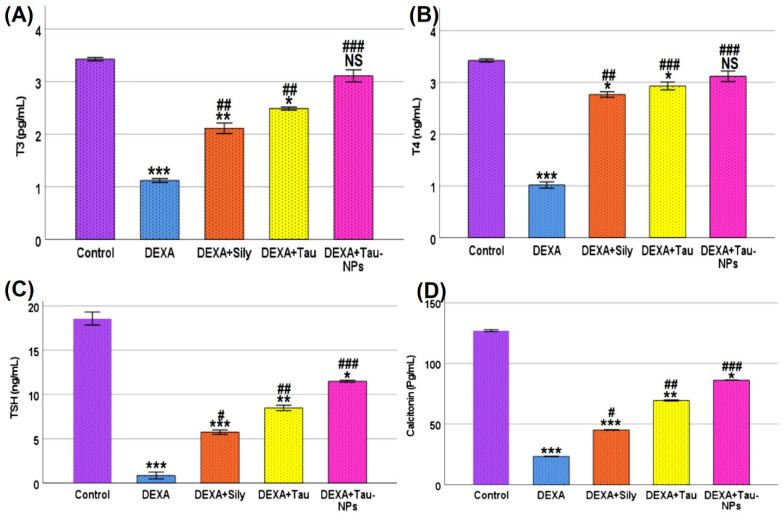
Effect of taurine and taurine-loaded nanoparticles on thyroid hormonal profile and calcitonin levels in dexamethasone-treated rats. (**A**) Serum triiodothyronine (T3), (**B**) serum thyroxine (T4), (**C**) serum thyroid-stimulating hormone (TSH), and (**D**) serum calcitonin levels in control and treated groups. Data are presented as Mean ± SD (*n* = 8). Statistical analysis was performed using one-way ANOVA followed by Tukey’s post hoc test. *** *p* < 0.001, ** *p* < 0.01, and * *p* < 0.05 indicate significant differences compared with the control group. #, ##, and ### indicate significant differences compared with the dexamethasone (DEXA) group at *p* < 0.05, *p* < 0.01, and *p* < 0.001, respectively. NS: non-significant compared with the control group.

As illustrated in Figure 3, dexamethasone administration induced a significant disturbance in thyroid function, as evidenced by a marked decrease in serum T3, T4, and TSH, in addition to a significant reduction in serum calcitonin levels compared with the control group (*p* < 0.001). These findings indicate suppression of the hypothalamic–pituitary–thyroid (HPT) axis following dexamethasone exposure.

Co-treatment with silymarin significantly improved thyroid hormonal status, leading to partial restoration of serum T3 and T4 levels along with a significant elevation of TSH and calcitonin levels compared with the dexamethasone group (*p* < 0.05–0.01). Taurine administration exerted a stronger corrective effect, showing further normalization of thyroid hormones and calcitonin, accompanied by a more pronounced recovery of TSH levels.

Notably, taurine-loaded nanoparticles (Tau-NPs) exhibited the most potent protective effect, restoring serum T3, T4, TSH, and calcitonin levels to values statistically not significantly comparable to those of the control group. These results underscore the superior efficacy of taurine nanoformulation in counteracting dexamethasone-induced suppression of thyroid function.

### 2.6. Serum Protein Profile

**Figure 4:**
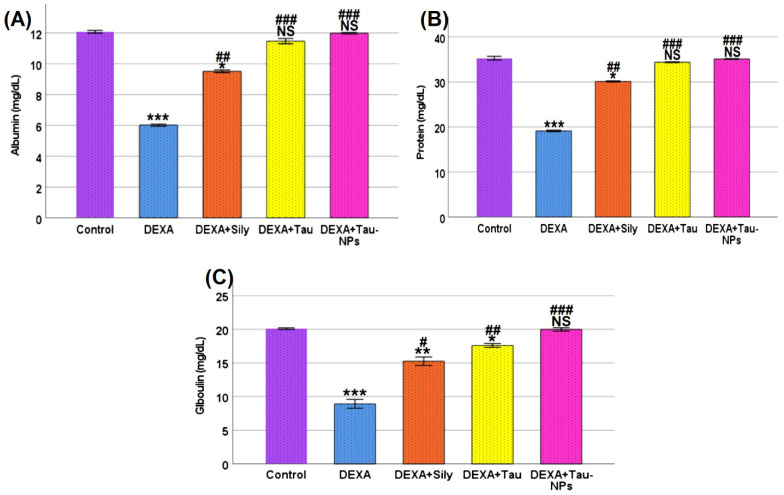
Effect of taurine and taurine-loaded nanoparticles on serum protein profile in dexamethasone-treated rats. (**A**) Serum albumin levels, (**B**) Total protein levels, (**C**) Serum globulin levels. Data are presented as Mean ± SD (*n* = 8). Statistical analysis was performed using one-way ANOVA followed by Tukey’s post hoc test. *** *p* < 0.001, ** *p* < 0.01, and * *p* < 0.05 indicate significant differences compared with the control group. #, ##, and ### indicate significant differences compared with the dexamethasone (DEXA) group at *p* < 0.05, *p* < 0.01, and *p* < 0.001, respectively. NS: non-significant compared with the control group.

As illustrated in Figure 4, dexamethasone administration caused a significant reduction in serum albumin, total protein, and globulin levels compared with the control group (*p* < 0.001), indicating marked impairment of protein synthesis and metabolic homeostasis.

Co-treatment with silymarin significantly ameliorated the dexamethasone-induced decline in serum albumin, total protein, and globulin levels, showing partial restoration compared with the DEXA group (*p* < 0.05–0.01). Taurine treatment exerted a more pronounced protective effect, leading to further improvement in all measured protein parameters.

Notably, taurine-loaded nanoparticles (Tau-NPs) demonstrated the strongest corrective efficacy, restoring serum albumin, total protein, and globulin levels to values statistically not significantly comparable to those of the control group. These findings highlight the superior role of taurine nanoformulation in counteracting dexamethasone-induced protein metabolism disturbances.

### 2.7. Histopathological Evaluation of Lung Tissue

**Figure 5:**
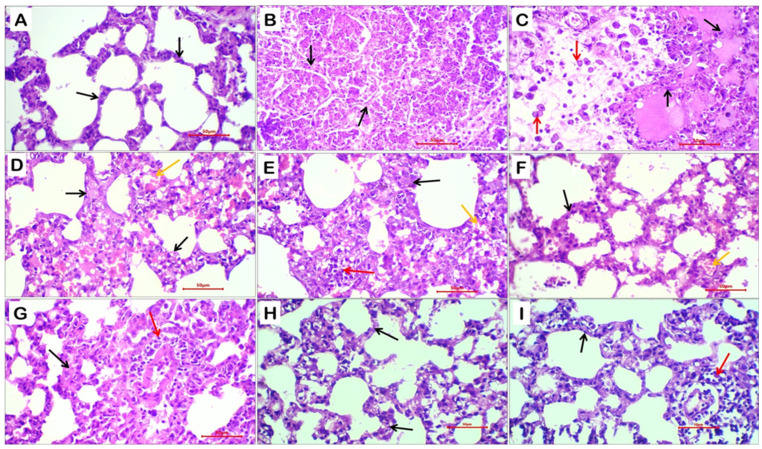
Representative photomicrographs of lung tissue sections from different experimental groups: control non-treated rats (**A**), dexamethasone-treated rats (**B**,**C**), dexamethasone + silymarin-treated rats (**D**,**E**), dexamethasone + taurine-treated rats (**F**,**G**), and dexamethasone + taurine nanoparticles-treated rats (**H**,**I**). Black arrows indicate alveolar walls, red arrows denote inflammatory cell infiltration, and yellow arrows indicate congested capillaries and/or interstitial tissue hemorrhage. Sections were stained with hematoxylin and eosin (H&E); original magnification ×400.

Administration of dexamethasone induced marked histopathological damage in lung tissue compared with the control non-treated rats. Histological examination of lung sections from the control group (Figure 5A) revealed normal pulmonary architecture with intact alveolar septa and wide, clear alveolar spaces. No evidence of alveolar septal thickening, edema, capillary congestion, or interstitial hemorrhage was observed.

In contrast, lung sections from dexamethasone-treated rats exhibited severe pathological alterations characterized by multiple foci of tissue necrosis, extensive destruction of alveolar septa, and collapse with obliteration of alveolar spaces, accompanied by marked interstitial hemorrhage (Figure 5B,C). Areas adjacent to necrotic regions showed pronounced thickening and edema of alveolar septa, congested capillaries, and intense inflammatory cell infiltration, predominantly macrophages, plasma cells, and neutrophils.

Co-administration of silymarin with dexamethasone resulted in partial attenuation of lung injury, as evidenced by a reduction in the extent of necrosis. However, noticeable histopathological abnormalities persisted, including frequent vascular congestion, interstitial hemorrhage, and focal inflammatory cell infiltration (Figure 5D,E).

Conversely, taurine co-treatment effectively prevented dexamethasone-induced pulmonary necrosis and markedly alleviated interstitial hemorrhage and inflammatory responses in the surrounding lung tissue (Figure 5F,G). Notably, treatment with taurine-loaded nanoparticles exhibited the most pronounced protective effect, restoring near-normal lung histoarchitecture. Lung sections from this group showed preserved alveolar structures with clear, patent alveolar spaces, minimal focal thickening of alveolar septa, and only mild patchy perivascular inflammatory infiltration, with complete absence of necrotic lesions (Figure 5H,I).

### 2.8. Histopathological Evaluation of Thyroid Tissue

**Figure 6:**
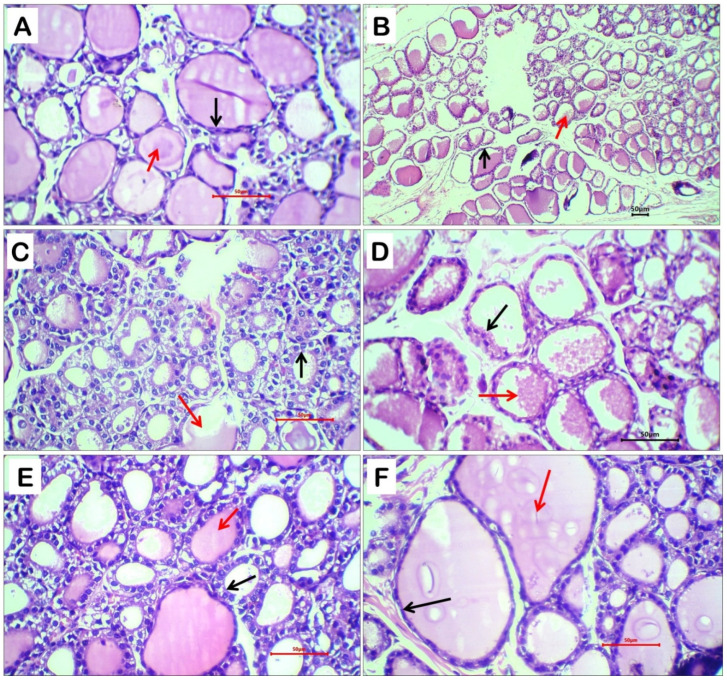
Representative photomicrographs of thyroid gland sections from different experimental groups: negative control rats (**A**), dexamethasone-treated rats (**B**,**C**), dexamethasone + silymarin-treated rats (**D**), dexamethasone + taurine-treated rats **(E),** and dexamethasone + taurine nanoparticles-treated rats (**F**). Black arrows indicate the epithelial lining of thyroid follicles, while red arrows denote the colloid content within the follicular lumen. Sections were stained with hematoxylin and eosin (H&E); original magnification ×100 for panel B and ×400 for all other panels.

Rats treated with dexamethasone exhibited marked thyroid hyperplastic changes compared with the negative control group. Histological examination of thyroid tissue from control rats (Figure 6A) revealed preserved thyroid architecture with variably sized follicles. These follicles were lined by a single layer of flat to low cuboidal thyrocytes and filled with dense eosinophilic colloid material, indicating a normal functional state.

In contrast, thyroid sections from dexamethasone-treated rats showed prominent hyperplastic alterations characterized by proliferation of small- to medium-sized follicles lined predominantly by cuboidal thyrocytes, along with depletion or pallor of intraluminal eosinophilic colloid material (Figure 6B,C). These features are indicative of enhanced colloid resorption and increased thyroidal activity associated with thyroxine synthesis.

Treatment with silymarin or taurine partially attenuated dexamethasone-induced thyroid alterations. In both groups, thyroid follicles were mainly lined by cuboidal epithelial cells with reduced or pale colloid content, reflecting moderate improvement but persistence of functional activation (Figure 6D,E).

Notably, concurrent administration of taurine-loaded nanoparticles resulted in marked regression of thyroid hyperplastic changes. Thyroid follicles displayed partial restoration of normal architecture, with focal lining by flattened thyrocytes and reappearance of dense eosinophilic colloid material within the follicular lumen (Figure 6F), indicating substantial recovery toward the normal thyroid histological pattern.

### 2.9. Expression of Myeloperoxidase (MPO)

**Figure 7:**
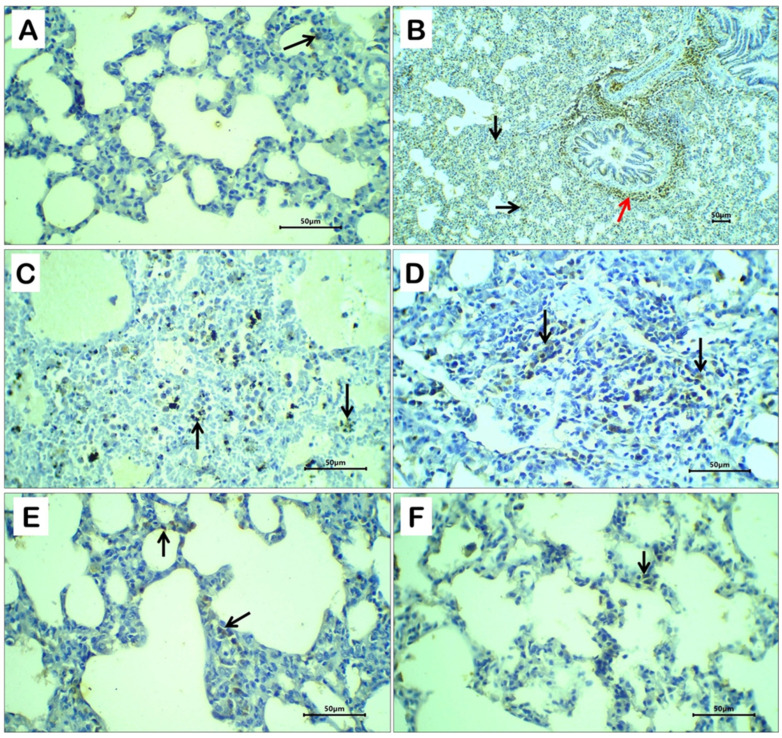
Immunohistochemically stained lung sections of control non-treated rats (**A**), dexamethasone-treated rats (**B**,**C**), dexamethasone + silymarin-treated rats (**D**), dexamethasone + taurine-treated rats (**E**), and dexamethasone + taurine nanoparticles-treated rats (**F**). Black arrows indicate MPO-positive inflammatory cells within the alveolar septa, while red arrows indicate MPO-positive peribronchial inflammatory cells. Immunostained sections; magnification ×100 for panel B and ×400 for all other panels.

Immunohistochemical expression of myeloperoxidase (MPO), a tissue marker indicative of inflammatory response, was demonstrated as brown cytoplasmic staining in inflammatory cells. Lung tissue sections from negative control rats showed only a few scattered MPO-positive cells (Figure 7A). In contrast, rats treated with dexamethasone exhibited marked and diffuse MPO immunoreactivity in inflammatory cells within the alveolar septa, peribronchial regions (Figure 7B), as well as in inflammatory cells infiltrating necrotic areas (Figure 7C), indicating a pronounced inflammatory response.

Co-treatment with silymarin or taurine markedly attenuated the dexamethasone-induced inflammatory reaction, as evidenced by a patchy and reduced MPO expression in inflammatory cells localized mainly within the alveolar septa (Figure 7D and E, respectively). Notably, treatment with taurine nanoparticles exerted a more potent anti-inflammatory effect, demonstrated by minimal residual MPO-positive inflammatory cells within the alveolar septa (Figure 7F).

## 3. Discussion

The present study provides comprehensive evidence on the protective potential of taurine and taurine-loaded chitosan nanoparticles (Tau–CS NPs) against dexamethasone-induced systemic toxicity, integrating hematological, biochemical, oxidative, histopathological, and immunohistochemical assessments.

The present physicochemical characterization of taurine-loaded chitosan nanoparticles (Tau–NPs) confirms not only successful fabrication but also the generation of a highly promising drug delivery system with distinct advantages over free taurine. Transmission electron microscopy revealed predominantly spherical to quasi-spherical nanoparticles with sizes ranging approximately between 11.4 and 60.7 nm and a narrow size distribution, indicating robust control over synthesis and high morphological uniformity, features strongly associated with enhanced cellular uptake and biological interactions in nanoscale drug delivery systems (size < 100 nm is optimal for endocytosis and tissue penetration). The smooth surface morphology and limited aggregation further support effective encapsulation and colloidal stability, which are critical determinants of nanocarrier performance in vivo [13].

Encapsulation efficiency and drug loading capacity are among the most important metrics of nanocarrier efficacy. The current Tau–NPs exhibited an encapsulation efficiency (EE%) of 98.2% and a high drug loading capacity (DLC%) of 50.36%. Such high EE underscores the strong electrostatic and hydrogen bonding interactions between taurine and the chitosan matrix, as suggested by the FTIR spectral shifts, reduced band intensities, and peak broadening associated with –NH and –OH groups. This level of encapsulation compares favorably with recent chitosan-based nanocarrier systems, which reported EE values ranging from ~70% to >90% depending on formulation and drug characteristics, demonstrating that optimized chitosan matrices can efficiently retain both hydrophilic and amphipathic molecules [14].

The significant advantage of chitosan as a nanocarrier lies not only in its innate biocompatibility and biodegradability, features that are highly desirable for clinical translation and are recognized by regulatory authorities for safety, but also in its mucoadhesive and permeability-enhancing properties. Chitosan’s cationic amino groups interact electrostatically with negatively charged mucosal surfaces, prolonging retention at absorption sites and facilitating paracellular transport across epithelial barriers, which can markedly improve bioavailability compared to free drug forms [15]. Indeed, enhanced drug absorption has been demonstrated with chitosan nanoparticles in diverse drug delivery contexts, with reports showing multiple-fold improvements in systemic availability and sustained release profiles relative to non-nanoparticulate formulations. Such improvements are attributed to controlled release kinetics, protection from early degradation, and modulation of biological barriers [16].

In addition, Tau–CS NPs, by virtue of their nanoscale size and surface properties, likely benefit from the enhanced permeation and retention (EPR) effect, which facilitates selective accumulation in inflamed or damaged tissues where vascular permeability is increased. This passive targeting phenomenon, well documented for polymeric nanocarriers, contrasts with the rapid systemic clearance typical of small free molecules and could underlie the superior in vivo efficacy observed in later biological assessments (e.g., hematological and oxidative stress endpoints). Such an enhanced therapeutic index is consistent with reports showing that polymeric nanoparticles can improve the pharmacokinetic profile and tissue distribution of encapsulated agents, leading to heightened efficacy at lower systemic doses while mitigating off-target effects [13].

Moreover, the intrinsic biodegradability of chitosan, degraded enzymatically into non-toxic glucosamine and oligosaccharides, provides a biodegradation pathway that minimizes long-term accumulation and toxicity, a key consideration for repeated or chronic therapeutic applications. Compared with free taurine, which is rapidly cleared and subject to systemic metabolism, the nanoencapsulated form ensures prolonged availability, potentially enhancing the duration of antioxidant and anti-inflammatory effects at target tissues.

Although TEM confirmed nanoparticle morphology and dry-state size, hydrodynamic diameter and colloidal dispersion in biological media were not evaluated using dynamic light scattering (DLS), which represents a limitation for complete physicochemical characterization.

Dexamethasone exposure elicited pronounced hematological disruptions, manifested by significant hemoglobin reduction alongside leukocytosis, neutrophilia, and relative lymphopenia, eosinopenia, and monocytopenia. These changes reflect a stress-induced immunological imbalance and inflammatory perturbation, consistent with glucocorticoid-mediated suppression of erythropoiesis and redistribution of leukocyte subpopulations [17]. The decrease in hemoglobin may also be attributable to dexamethasone’s known catabolic effects on bone marrow activity and erythroid progenitor cells, while neutrophilia and lymphopenia represent hallmark glucocorticoid-driven shifts in circulating leukocyte kinetics [18]. Co-treatment with silymarin partially ameliorated these hematological derangements, aligning with its reported antioxidant and immunomodulatory properties that moderately counteract steroid-induced marrow suppression and leukocyte dysregulation [19].

Taurine administration produced more substantial improvement in leukocyte differentials and hemoglobin levels compared to silymarin. Taurine’s involvement in membrane stabilization, modulation of osmotic balance, and regulation of neutrophil activation likely underpins this effect, as taurine has been shown to attenuate inflammatory leukocyte responses and protect erythrocytes from oxidative damage [20,21]. However, the most compelling restoration of hematological homeostasis was observed with Tau-NPs, which normalized hemoglobin and leukocyte indices to values that did not significantly differ from control. The superior efficacy of the nanoparticulate formulation likely stems from enhanced bioavailability, improved cellular uptake, and a sustained release profile that prolongs systemic taurine exposure relative to the free compound [22,23]. Nanocarrier delivery systems, particularly chitosan-based nanoparticles, have been repeatedly demonstrated to enhance the pharmacokinetic properties and therapeutic index of encapsulated agents by protecting them from rapid metabolic degradation and facilitating targeted tissue interaction [24]. This enhanced pharmacodynamic profile likely contributes to the observed mitigation of dexamethasone-induced hematological toxicity by Tau-NPs, positioning them as a more effective strategy for preserving hematopoietic and immune parameters under steroid-induced stress.

Dexamethasone administration induced profound oxidative stress in lung tissue, as evidenced by significant reductions in catalase (CAT), superoxide dismutase (SOD), and reduced glutathione (GSH), accompanied by elevated malondialdehyde (MDA) levels, indicating severe lipid peroxidation. These findings align with the established role of glucocorticoids in promoting reactive oxygen species (ROS) generation and disrupting pulmonary redox homeostasis, thereby impairing antioxidant defenses [25]. The lung is particularly vulnerable to oxidative damage due to its high oxygen exposure and extensive vascularization, which amplify ROS-mediated oxidation of membranes and proteins.

Co-treatment with silymarin partially attenuated dexamethasone-induced oxidative injury, as indicated by the partial restoration of antioxidant enzyme activities and reduced MDA accumulation. Silymarin, a flavonolignan extract from *Silybum marianum*, has well-documented antioxidant properties, including free radical scavenging, enhancement of endogenous antioxidant systems, and inhibition of lipid peroxidation, which are central to its protective actions in oxidative stress models [26]. Despite these protective actions, silymarin’s effects did not fully restore all oxidative markers to control levels, indicating only partial mitigation of the severe steroid-induced redox imbalance.

Taurine treatment resulted in a more marked improvement in lung antioxidant status than silymarin, with higher recovery of CAT, SOD, and GSH activities and a greater reduction in MDA levels. Taurine’s antioxidant capacity has been attributed to multiple mechanisms, including stabilization of cell membranes, modulation of calcium homeostasis, and formation of taurine chloramine, which attenuates ROS production and inflammatory responses [27,28]. These mechanisms enhance cellular resilience to oxidative insult and support redox balance more effectively than nonspecific antioxidants alone.

Most notably, taurine-loaded chitosan nanoparticles (Tau-NPs) demonstrated the most potent antioxidant effectiveness. Tau-NPs restored antioxidant enzyme activities and GSH content to values comparable with control, and markedly suppressed lipid peroxidation, as evidenced by near-normal MDA levels. The superior efficacy of Tau-NPs over free taurine can be largely attributed to the physicochemical advantages conferred by the chitosan nanocarrier. Chitosan is a biodegradable, biocompatible polymer with mucoadhesive and permeability-enhancing properties, widely used as a drug delivery vehicle to improve bioavailability and sustained release of encapsulated agents [29]. Encapsulation protects taurine from rapid degradation and allows for a controlled and prolonged interaction with lung tissues, which enhances intracellular uptake and potent antioxidant activity beyond that achievable with free taurine.

Our results demonstrate that although both silymarin and free taurine confer measurable protection against dexamethasone-induced pulmonary oxidative stress, nanoformulated taurine delivered via chitosan nanoparticles provides superior and comprehensive antioxidant defense. The enhanced performance of Tau-NPs highlights the therapeutic promise of nanocarrier systems in mitigating oxidative injury in glucocorticoid-exposed lung tissues.

Similarly, dexamethasone exposure elicited significant oxidative and inflammatory disturbances in thyroid tissue, as demonstrated by increased malondialdehyde (MDA) and nitric oxide (NO) levels, concomitant with marked depletion of intrinsic antioxidant defenses such as superoxide dismutase (SOD), reduced glutathione (GSH), and quinone reductase (QR), alongside elevated gamma-glutamyl transferase (GGT) activity. These biochemical perturbations reflect an imbalance between prooxidant generation and antioxidant capacity, consistent with the thyroid’s inherent susceptibility to ROS due to its high mitochondrial activity and continuous hydrogen peroxide production during hormone synthesis, making it particularly vulnerable to oxidative damage under stress conditions. Excessive ROS and NO can interact with lipids and proteins, causing peroxidative injury and nitrosative stress that compromise cellular integrity and detoxification pathways, as evidenced by elevated GGT, a surrogate marker for cellular stress and impaired detoxification [30].

Co-treatment with silymarin partially attenuated these alterations, as shown by significant reductions in MDA, NO, and GGT levels and moderate restoration of antioxidant indices. Silymarin, a flavonolignan complex extracted from *Silybum marianum*, has been extensively documented for its antioxidant and anti-inflammatory actions in diverse tissues, including suppression of lipid peroxidation and upregulation of endogenous antioxidant enzyme activities. Specifically, silymarin and its constituents have been shown to scavenge free radicals, inhibit reactive species formation, and modulate redox-sensitive signaling pathways [31]. However, the partial nature of this effect in our model suggests that silymarin alone may be insufficient to fully counteract the profound oxidative insult induced by high-dose glucocorticoids.

Administration of taurine conferred a more pronounced protective effect, significantly improving oxidative balance and reducing inflammatory burden in thyroid tissue. Taurine’s antioxidant actions have been corroborated across numerous experimental models, where it stabilizes cellular membranes, preserves glutathione pools, and enhances antioxidant enzyme activities, while simultaneously mitigating ROS-mediated damage and inflammatory signaling cascades. Mechanistically, taurine is recognized for its ability to attenuate lipid peroxidation, limit oxidative DNA damage, and modulate redox-sensitive pathways that underlie inflammation and detoxification impairment, supporting its cytoprotective role in stress-challenged tissues [7].

Notably, taurine-loaded nanoparticles (Tau-NPs) exhibited the most potent efficacy, markedly restoring antioxidant and detoxification parameters toward control levels. The enhanced effect of the nanoformulation likely arises from improved bioavailability and targeted delivery, which facilitate greater intracellular uptake and sustained presence at the site of injury compared with free taurine. Nanocarrier systems, such as chitosan nanoparticles, have been shown to protect encapsulated bioactives from premature degradation, improve tissue penetration, and prolong therapeutic activity, thereby amplifying their antioxidant and anti-inflammatory potential [32]. This sustained redox modulation likely underpins the normalization of GSH, reduction in MDA and NO, and improved QR and GGT activity observed with Tau-NPs, indicating robust restoration of thyroid homeostasis and detoxification capacity.

Collectively, these data indicate that while both silymarin and free taurine provide measurable protection against glucocorticoid-induced thyroid oxidative stress and inflammatory injury, taurine nanoformulation markedly enhances these effects, highlighting the therapeutic promise of nanocarrier-mediated antioxidant delivery in mitigating endocrine oxidative damage and functional impairment.

Dexamethasone administration in the current study resulted in a pronounced suppression of the hypothalamic–pituitary–thyroid (HPT) axis, manifesting as significant reductions in circulating T3, T4, TSH, and calcitonin levels. These alterations are consistent with the well-documented suppressive effects of glucocorticoids on thyroid function, where dexamethasone decreases endogenous TSH secretion and reduces peripheral conversion of T4 to the more active T3, leading to transient decreases in serum thyroid hormones (Turner syndrome and stress studies; dexamethasone suppresses TSH and T3 in humans) [33]. Suppression of TSH by glucocorticoids occurs at both the hypothalamic and pituitary levels, attenuating thyrotropin release and shifting the setpoint of the HPT feedback loop, which explains the concurrent decreases in T3 and T4 observed in the dexamethasone group [34]. The observed reduction in serum calcitonin further reflects the global inhibitory impact of dexamethasone on thyroid secretory activity, as calcitonin release is closely tied to thyroid C-cell function and responsive to alterations in systemic endocrine signaling.

Co-treatment with silymarin elicited a significant improvement in thyroid hormonal profile, with partial restoration of T3, T4, TSH, and calcitonin levels toward control values. The ability of silymarin to modulate endocrine responses likely stems from its potent antioxidant and anti-inflammatory actions, which mitigate oxidative damage and facilitate recovery of endocrine cell function under stress conditions. By scavenging free radicals and attenuating stress-induced signaling pathways, silymarin may relieve inhibitory feedback on the HPT axis, indirectly permitting normalization of hormone synthesis and release. However, this corrective effect remained incomplete, suggesting a limited capacity of silymarin alone to fully reverse profound glucocorticoid-induced hormonal suppression.

Taurine administration exerted a more substantial corrective effect on thyroid hormones and calcitonin, achieving greater normalization compared with silymarin. Taurine’s multifaceted biological roles—particularly its ability to stabilize cellular membranes, regulate intracellular calcium homeostasis, and modulate redox signaling—can support thyroid follicular cell integrity and enhance the synthetic capacity for thyroid hormones under stress conditions. In experimental endocrine models, taurine supplementation has been shown to restore thyroidal disturbances and improve circulating hormone levels, likely via attenuation of oxidative/nitrosative stress and preservation of HPT axis responsiveness [8,35]. These actions may help sustain adequate thyroid hormone production and secretion in the face of glucocorticoid-mediated inhibition.

Notably, taurine-loaded nanoparticles exhibited the most potent protective efficacy, fully restoring T3, T4, TSH, and calcitonin levels to values that were not significantly different from those of control animals. The nanoparticle formulation enhances taurine’s bioavailability and tissue penetration, promoting sustained release and more efficient intracellular delivery to target endocrine cells compared with free taurine. Nanocarrier systems such as chitosan nanoparticles are known to improve the pharmacokinetic and pharmacodynamic profiles of encapsulated agents, protecting them from rapid systemic clearance and enabling prolonged interaction with endocrine tissues, which likely underlies the superior normalization of thyroid hormone profiles observed with Tau-NPs. Additionally, improved antioxidant and anti-inflammatory effects through enhanced cellular uptake may have synergistically alleviated HPT axis suppression, facilitating full recovery of thyroid hormonal output.

Dexamethasone treatment caused a significant decline in serum albumin, total protein, and globulin levels, reflecting a disturbance in protein metabolism and metabolic homeostasis. Glucocorticoids like dexamethasone are known to induce a catabolic state characterized by enhanced proteolysis and impaired protein turnover in vivo, which contributes to protein wasting and negative nitrogen balance [8]. This catabolic action can reduce circulating protein pools either through increased breakdown or alterations in synthesis and transport, consistent with clinical observations of lowered total protein and albumin in conditions of chronic glucocorticoid excess [36].

Co-treatment with silymarin partially improved serum protein parameters, likely through its antioxidative and hepatoprotective effects that support hepatic protein synthesis under stress conditions; however, these improvements were modest compared with those seen with taurine. Taurine administration produced a more pronounced amelioration of protein disturbances, in line with evidence that taurine can modulate cellular metabolism and support protein homeostasis in experimental models. For example, taurine supplementation has been shown to affect whole-body protein turnover and accretion, indicating a regulatory influence on protein metabolism through improved amino acid utilization and metabolic signaling [37].

Notably, the strongest normalization of albumin, total protein, and globulin levels was observed in rats treated with taurine-loaded nanoparticles (Tau-NPs). The enhanced efficacy of Tau-NPs relative to free taurine can be mechanistically attributed to improved bioavailability, protection from rapid degradation, and sustained release of taurine at target sites, which together potentiate its metabolic effects more effectively than the free form. Nanocarrier systems such as chitosan nanoparticles are widely recognized for improving the pharmacokinetic and pharmacodynamic profiles of encapsulated agents, prolonging systemic exposure, and facilitating enhanced cellular uptake, thereby enabling more efficient restoration of metabolic balance (general nanocarrier drug delivery reviews). Collectively, these findings highlight the superior role of taurine nanoformulation in counteracting dexamethasone-induced disruptions in protein metabolism and support its potential therapeutic utility in reducing steroid-related metabolic derangements.

Taurine exerts its biological effects through multiple interconnected molecular pathways. Its antioxidant activity is primarily mediated by direct scavenging of reactive oxygen species (ROS) and the formation of taurine chloramine (TauCl), a stable compound with potent anti-inflammatory properties [38]. Taurine also modulates intracellular calcium homeostasis, thereby stabilizing mitochondrial function and preventing apoptosis induced by oxidative stress [39]. Furthermore, it downregulates pro-inflammatory signaling pathways, including NF-κB activation, leading to reduced expression of cytokines such as TNF-α and IL-6 [40,41]. In addition, taurine contributes to membrane stabilization and osmotic regulation, which collectively support cellular integrity under toxic stress conditions [42]. These pleiotropic mechanisms may explain its protective role against dexamethasone-induced pulmonary and thyroid dysfunction observed in the present study.

Histopathological examination of lung and thyroid tissues provided compelling morphological evidence supporting the biochemical and functional findings. Dexamethasone administration induced pronounced pulmonary damage, characterized by alveolar septal necrosis, interstitial hemorrhage, edema, and dense inflammatory infiltration, reflecting severe oxidative and inflammatory injury. Similarly, thyroid sections exhibited hyperplastic changes, with proliferation of cuboidal thyrocytes and depletion of colloid content, indicative of enhanced thyroidal activity and structural stress. Immunohistochemical analysis further confirmed these findings, as marked MPO expression highlighted the extensive inflammatory infiltration in lung tissue. Co-treatment with silymarin or free taurine partially mitigated these histopathological alterations, consistent with their known antioxidant and anti-inflammatory properties. Remarkably, taurine-loaded nanoparticles (Tau-NPs) demonstrated the most pronounced protective effect, preserving lung alveolar architecture, reducing inflammatory cell infiltration, and restoring thyroid follicular morphology toward normal, reflecting the enhanced bioavailability, targeted delivery, and sustained release of taurine provided by the nanocarrier system. These results underscore the superior therapeutic potential of taurine nanoformulations in ameliorating steroid-induced tissue damage, aligning with recent studies demonstrating improved tissue protection via nanoparticle-mediated delivery of bioactive compounds [13].

Despite the promising findings of the present study, several limitations should be acknowledged. First, this investigation was conducted using a rodent model, which may not fully replicate human physiological and pathological responses, thereby limiting direct clinical translation. Second, although a broad range of biochemical, hematological, hormonal, and histopathological parameters were evaluated, the long-term effects of taurine nanoformulations and their precise molecular signaling mechanisms were not comprehensively investigated. In particular, deeper validation of pathways related to oxidative stress, inflammation, apoptosis, and endocrine regulation remains necessary. Third, the study examined only a single taurine dose and nanoparticle formulation, without assessing dose–response relationships, pharmacokinetics, or biodistribution patterns, which are essential for optimizing therapeutic efficacy and safety. Fourth, nanoparticle characterization was primarily based on transmission electron microscopy (TEM), which provides dry-state morphological and size evaluation only. Comprehensive physicochemical characterization, including dynamic light scattering (DLS) for hydrodynamic diameter, polydispersity index (PDI), colloidal stability in biological media, and long-term post-lyophilization stability assessments, was beyond the scope of this study. Therefore, future work should further evaluate storage stability, encapsulation integrity, release kinetics, and formulation robustness under physiological and prolonged storage conditions. Additionally, while histopathological examination revealed clear qualitative tissue alterations, semi-quantitative or blinded scoring systems were not applied, which may limit the precision of comparative tissue analysis. Incorporating standardized histopathological scoring approaches in future studies would strengthen objective tissue evaluation and improve correlation with biochemical outcomes. Collectively, future investigations should focus on advanced mechanistic studies, expanded nanoparticle characterization, long-term safety assessments, and validation in higher-order preclinical models to enhance translational applicability.

## 4. Materials and Methods

### 4.1. Materials and Drugs

Chitosan (low molecular weight), sodium tripolyphosphate (TPP), taurine, silymarin, and pharmaceutical-grade dexamethasone were purchased from Glentham Life Sciences Ltd. (Corsham, UK). All chemicals and reagents were of analytical grade and were used as received without further purification.

### 4.2. Reagents and Kits

All biochemical, oxidative stress, inflammatory, detoxification, and hormonal parameters were quantified using commercially available assays and ELISA kits, strictly following the manufacturers’ instructions.

Serum thyroid function parameters, including thyroid-stimulating hormone (TSH), total triiodothyronine (T3), and total thyroxine (T4), were determined using rat ELISA kits purchased from MyBioSource, Inc. (San Diego, CA, USA). Serum-free triiodothyronine (FT3) and free thyroxine (FT4) levels were measured by ELISA using rat-specific kits obtained from Elabscience Biotechnology Co., Ltd. (Houston, TX, USA) (Cat. Nos. MBS267920, MBS3808254, and MBS3808033, respectively). Serum calcitonin (CT) concentrations were quantified using a Rat Calcitonin ELISA Kit (Cat. No. E-EL-R0047) supplied by Elabscience Biotechnology Co., Ltd.

Oxidative stress, inflammatory, and detoxification markers were assessed using ELISA kits purchased from MyBioSource, Inc. (San Diego, CA, USA), including quinone reductase (QR; Cat. No. MBS7606601, expressed as pg/mL), nitric oxide (NO; Cat. No. MBS2604161), and gamma-glutamyl transpeptidase (GGT; Cat. No. MBS9343646).

Antioxidant defense biomarkers were determined in lung and thyroid tissue homogenates using colorimetric assay kits obtained from Nanjing Jiancheng Bio-Technology Co., Ltd. (Nanjing, China). These included superoxide dismutase (SOD; Cat. No. A001-3), malondialdehyde (MDA; Cat. No. A003-1), reduced glutathione (GSH; Cat. No. A006-2-1), and catalase (CAT; Cat. No. A007-1-1).

Inflammatory activity was further evaluated by measuring myeloperoxidase (MPO) levels using a Rat MPO ELISA Kit purchased from Abcam (Cambridge, UK) (Cat. No. ab285308).

All kits used in the present study were validated for experimental research, exhibited high sensitivity and specificity, and ensured accurate and reliable quantification of the investigated parameters.

### 4.3. Preparation of Taurine Nanoformulation

Taurine-loaded chitosan nanoparticles (Tau NPs) were prepared at the Faculty of Science, Sohag University, Egypt, using a modified ionic gelation method based on previously described protocols for chitosan nanoparticle formation. Briefly, taurine was dissolved in a minimal volume of dimethyl sulfoxide (DMSO) under continuous magnetic stirring. Separately, chitosan was dissolved in 1% (*v*/*v*) acetic acid at room temperature and diluted with distilled water. The taurine solution was then added to the chitosan solution under magnetic stirring (500 rpm) [43]. Nanoparticle formation was induced by the dropwise addition of sodium tripolyphosphate (TPP) solution at a controlled rate using a burette, while maintaining continuous stirring, allowing the electrostatic interaction between the positively charged amino groups of chitosan and the negatively charged TPP to form nanoparticles (ionic gelation) [44].

The resulting suspension was stirred for an additional 30 min to ensure stable nanoparticle formation. Nanoparticles were collected by ultracentrifugation (Hanil Micro 17TR centrifuge—HE5) at 17,000 rpm and 4 °C for 30 min, followed by lyophilization and storage at 4 °C until further use. The prepared taurine nanoparticles were characterized for physicochemical properties, including particle size distribution, morphology by transmission electron microscopy (TEM), Fourier transform infrared spectroscopy (FT-IR), entrapment efficiency (EE), and drug loading capacity (DLC). Residual DMSO was minimized through ultracentrifugation and lyophilization; however, direct solvent quantification was not performed.

Following nanoparticle synthesis and purification, taurine-loaded chitosan nanoparticles were lyophilized to improve storage stability and facilitate subsequent experimental use. The lyophilized nanoparticles were stored at 4 °C in sealed containers until administration. Before use, nanoparticles were freshly reconstituted under standardized conditions. Although lyophilization was employed to enhance formulation preservation, extended long-term physicochemical stability studies, including potential alterations in particle size distribution, morphology, and encapsulation efficiency after storage, were not comprehensively evaluated in the present study.

### 4.4. Evaluation and Characterization of Taurine Nanoparticles

The physicochemical characterization of taurine-loaded chitosan nanoparticles (Tau-NPs) was performed to evaluate particle size, size distribution, surface morphology, chemical interactions, encapsulation efficiency (EE), and drug loading capacity (DLC). The morphology, particle size, and size distribution of Tau-NPs were examined using transmission electron microscopy (TEM) operated at an accelerating voltage of 100 kV JEOL JEM-100 CXII (JEOL Ltd., Japan), allowing detailed visualization of nanoparticle shape, surface smoothness, and dispersion.

To accurately determine the concentration of free (non-encapsulated) taurine in the nanoparticle supernatant, a standard calibration curve was established using UV–visible spectrophotometry UviLine 9400 spectrophotometer (SECOMAM, Alès, France) at the maximum absorbance wavelength of taurine (λmax ≈ 210 nm). Standard taurine solutions were freshly prepared at concentrations ranging from 0 to 100 µg/mL in appropriate solvent under identical experimental conditions. The absorbance of each standard concentration was measured in triplicate, and a linear calibration curve was generated by plotting absorbance values against taurine concentrations. The method demonstrated excellent linearity over the tested concentration range, with a regression equation of y = 0.0098x + 0.0019 and a correlation coefficient (R^2^) of 1.000, confirming high analytical accuracy and reproducibility. Blank controls were included to exclude solvent or reagent interference. This validated calibration curve was subsequently used to quantify free taurine in the supernatant for the calculation of encapsulation efficiency (EE%) and drug loading capacity (DLC%).

Fourier transform infrared (FTIR) spectroscopy was employed to investigate the chemical structure of Tau-NPs and to identify potential interactions between taurine and the chitosan polymer matrix. FTIR spectra were recorded using an attenuated total reflectance Fourier transform infrared spectrometer (ATR-FTIR spectrometer (Alpha Bruker Platinum ATR; Bruker Optics GmbH, Ettlingen, Germany)). Characteristic absorption bands corresponding to specific functional groups of taurine and chitosan were analyzed to confirm successful nanoparticle formation and taurine encapsulation through ionic interactions.

UV–visible spectrophotometric analysis of free taurine was performed at its characteristic maximum absorbance wavelength (λmax ≈ 210 nm) using a validated calibration curve. Blank samples containing all formulation components except taurine were analyzed under identical conditions to exclude background interference from chitosan, tripolyphosphate, DMSO, or other excipients. Standard taurine solutions were measured in triplicate to confirm assay linearity, sensitivity, and reproducibility. The use of blank controls and comparative spectral analysis ensured analytical specificity and minimized potential interference, thereby allowing accurate quantification of non-encapsulated taurine for encapsulation efficiency determination.

The encapsulation efficiency (EE) and drug loading capacity (DLC) of Tau-NPs were determined after separating the nanoparticles from the suspension by ultracentrifugation using a Hanil Micro 17TR centrifuge (HE5) (Hanil Scientific Inc., Gimpo-si, Gyeonggi-do, South Korea) at 17,000 rpm and 4 °C for 30 min. The concentration of free (non-encapsulated) taurine in the supernatant was quantified using a UV–visible spectrophotometer at an appropriate wavelength based on taurine absorbance characteristics. EE and DLC were calculated using the following equations:

Encapsulation efficiency (%) = [(T − F)/T] × 100

Drug loading capacity (%) = [(T − F)/W] × 100

where F represents the amount of free taurine in the supernatant (mg), T is the total amount of taurine initially added to the chitosan solution (mg), and W denotes the total weight of the obtained nanoparticles (mg).

### 4.5. Experimental Animals

Healthy adult male Wistar rats weighing 180–200 g were used in the present study. All experimental procedures were conducted in accordance with the ethical guidelines approved by the Sohag Institutional Animal Care and Use Committee (Sohag-IACUC), Faculty of Science, Sohag University (protocol no.: SU-FS-6-26). Every effort was made to minimize animal discomfort and suffering throughout the experimental period. The animals were housed under controlled environmental conditions and acclimatized for two weeks before the start of the experiment at a temperature of 25 ± 2 °C, relative humidity of 65 ± 10%, with 11–13 air changes per hour, and a 12-h light/dark cycle. Rats were fed a standard laboratory pellet diet and were provided with free access to water ad libitum.

### 4.6. Experimental Design

After acclimatization, rats were randomly assigned to five experimental groups (*n* = 8 rats per group). Group size (*n* = 8 per group) was selected based on prior comparable in vivo toxicological studies and according to established resource equation and statistical power considerations for animal experimentation, which support this sample size as sufficient for detecting significant biochemical and histopathological differences while minimizing unnecessary animal use [45,46].

The experimental protocol was designed to assess the effects of dexamethasone alone and in combination with silymarin, taurine, and taurine nanoparticles. All treatments were administered once daily unless otherwise stated.

Control group: Received an equivalent volume of normal saline vehicle (2 mL/kg) throughout the experimental period.

Dexamethasone (DEXA) group: Rats received dexamethasone subcutaneously at a dose of 0.1 mg/kg body weight/day for 14 consecutive days to induce systemic toxicity, oxidative stress, and histopathological alterations. This dose and route have been widely used to establish dexamethasone-induced oxidative and tissue injury models in rats [47].

DEXA + Silymarin (Sily) group: Rats received dexamethasone as above (0.1 mg/kg, s.c., 14 days) plus silymarin administered orally by gavage at 100 mg/kg body weight/day for the same duration. The chosen silymarin dose is well documented for its antioxidant and cytoprotective effects in rodent models of steroid-induced oxidative stress [48].

DEXA + Taurine (Tau) group: Rats received dexamethasone (0.1 mg/kg, s.c., 14 days) plus taurine administered orally by gavage at 150 mg/kg body weight/day for 14 days. This taurine dose has been used experimentally to mitigate oxidative stress and inflammatory responses in rat models [49].

DEXA + Taurine nanoparticles (Tau-NPs) group: Rats received dexamethasone (0.1 mg/kg, s.c., 14 days) plus taurine nanoparticles equivalent to a taurine dose of 150 mg/kg body weight/day administered orally for 14 consecutive days. Nanoformulation dosing was calculated based on equivalent taurine content to ensure consistent exposure across delivery forms.

All treatments were administered at approximately the same time each day. The doses were selected based on previously published studies demonstrating reproducible induction of dexamethasone-related tissue toxicity and the protective efficacy of antioxidant compounds [48].

Silymarin was selected as a reference antioxidant compound due to its well-documented cytoprotective, anti-inflammatory, and antioxidant activities in glucocorticoid-induced oxidative injury models, allowing comparative evaluation of the therapeutic efficacy of taurine and taurine-loaded nanoparticles [26,50].

At the end of the experimental period, the animals were fasted overnight, anesthetized, and samples were collected for biochemical and histological analyses.

### 4.7. Blood Sampling for Hematological and Biochemical Analyses

At the end of the experimental period and following overnight fasting, rats were anesthetized under light anesthesia. Blood samples were collected via cardiac puncture using sterile disposable syringes. A portion of the collected blood was transferred immediately into ethylenediaminetetraacetic acid (EDTA)–containing tubes for hematological analysis, while the remaining blood was allowed to clot at room temperature. Serum was then separated by centrifugation at 3000 rpm for 15 min, aliquoted, and stored at −20 °C until biochemical and hormonal analyses were conducted.

Hematological parameters, including hemoglobin concentration (Hb), total leukocyte count, and differential leukocyte counts (neutrophils, lymphocytes, eosinophils, and monocytes), were determined using standard automated hematological methods.

All ELISAs were performed using a microplate reader with absorbance detection capability. The instrument used was a Thermo Fisher Multiskan GO microplate spectrophotometer (Thermo Fisher Scientific Oy, Vantaa, Finland), and measurements were recorded at the appropriate wavelength as specified in each assay kit protocol.

### 4.8. Determination of Pulmonary Oxidative Stress Markers

Lung tissues were excised immediately after sacrifice, rinsed with ice-cold saline, blotted dry, and accurately weighed. Each lung sample was homogenized in cold phosphate-buffered saline (PBS; pH 7.4, 0.01 mol/L) at a ratio of 1:4 (*w*/*v*) using a glass homogenizer under ice-cold conditions. The homogenates were centrifuged at 5000× *g* for 10 min at 4 °C, and the resulting supernatants were collected for biochemical analysis.

Pulmonary oxidative stress biomarkers, including reduced glutathione (GSH), superoxide dismutase (SOD), catalase (CAT), and malondialdehyde (MDA), were determined according to the manufacturer’s instructions using commercially available assay kits.

### 4.9. Determination of Inflammatory and Redox Markers in Lung Tissue

Lung tissue homogenates prepared as described above were also used to determine inflammatory and redox-related markers. Quinone reductase (QR) levels were evaluated as a cytoprotective and antioxidant enzyme. QR was measured using rat-specific ELISA kits following the manufacturer’s protocols, and the results were expressed per gram of lung tissue.

### 4.10. Determination of Thyroid Oxidative Stress and Biochemical Markers

Thyroid glands were carefully dissected, weighed, and homogenized in ice-cold phosphate-buffered saline (PBS; pH 7.4, 0.01 mol/L) at a ratio of 1:4 (*w*/*v*) using a glass homogenizer. The homogenates were centrifuged at 5000× *g* for 10 min at 4 °C, and the supernatants were collected for analysis. Thyroid oxidative stress parameters, including GSH, SOD, and MDA, as well as nitric oxide (NO) levels, were measured using validated rat-specific ELISA kits according to the manufacturers’ instructions. All assays were performed in triplicate.

### 4.11. Determination of Thyroid Function Parameters

Serum levels of thyroid-stimulating hormone (TSH), triiodothyronine (T3), thyroxine (T4), and calcitonin were measured using rat-specific enzyme-linked immunosorbent assay (ELISA) kits following the manufacturers’ instructions. Absorbance was measured using a microplate reader, and hormone concentrations were calculated from standard calibration curves provided with each kit.

### 4.12. Determination of Serum Protein Profile

Serum total protein and albumin concentrations were determined using commercially available diagnostic kits according to the manufacturers’ instructions. Serum globulin levels were calculated mathematically by subtracting albumin values from total protein concentrations. All measurements were performed using standard spectrophotometric methods.

### 4.13. Determination of Thyroid Hormonal Markers

Serum calcitonin levels were determined using rat-specific enzyme-linked immunosorbent assay (ELISA) kits following the manufacturer’s protocols. Absorbance was measured using a microplate reader, and hormone concentrations were calculated from standard curves provided with each kit.

### 4.14. Hematological Analysis

Whole blood samples collected in EDTA-containing tubes were used for hematological analysis. Hemoglobin concentration (Hb), total leukocyte count, and differential leukocyte counts, including neutrophils, lymphocytes, eosinophils, and monocytes, were determined using standard automated hematological techniques.

Hematological parameters were evaluated using an automated hematology analyzer. The system used was the Mindray BC-2800Vet, and the manufacturer’s operating procedures were followed to ensure the accuracy and reproducibility of the results.

### 4.15. Histopathological Studies

For histopathological examination of the different experimental groups, lung and thyroid tissue samples were collected immediately after sacrifice and fixed in 10% neutral buffered formalin for 24–36 h at room temperature. The fixed tissues were processed using standard histological procedures, including dehydration in ascending grades of ethyl alcohol, clearing in xylene, and embedding in paraffin wax at 70 °C. Paraffin blocks were sectioned at a thickness of 5 μm using a rotary microtome. The obtained sections were deparaffinized in xylene, rehydrated through descending grades of ethanol, and washed in running tap water. For routine histological evaluation, sections were stained with hematoxylin and eosin (H&E), where sections were immersed in hematoxylin for 7 min, washed thoroughly, and counterstained with eosin for 2 min. Subsequently, sections were dehydrated in ascending alcohol concentrations, cleared in xylene, and mounted using Dibutylphthalate Polystyrene Xylene (DPX). Histopathological examination was performed to assess structural alterations in lung tissue, including alveolar architecture, septal thickening, inflammatory cell infiltration, hemorrhage, and necrosis, as well as thyroid gland architecture, follicular size, epithelial cell morphology, and colloid content.

### 4.16. Immunohistochemical Studies

Immunohistochemical detection of myeloperoxidase (MPO) was performed to evaluate the inflammatory response in lung tissue. Four-micrometer-thick sections were cut from formalin-fixed, paraffin-embedded lung tissue blocks, deparaffinized in xylene, rehydrated through descending grades of ethanol, and washed in running tap water. Endogenous peroxidase activity was blocked by incubating the sections in 3% hydrogen peroxide (H_2_O_2_) for 10 min at room temperature. Antigen retrieval was carried out by heating the sections in 0.01 mol/L citrate buffer (pH 6.0) at 92 °C for 20 min. After cooling and washing with phosphate-buffered saline (PBS), tissue sections were incubated with a primary anti-myeloperoxidase (MPO) antibody for 1 h at room temperature. Following PBS washing, sections were incubated with an appropriate secondary antibody, then treated with a streptavidin–biotin complex, with PBS washes performed between each step. Immunoreactivity was visualized using diaminobenzidine (DAB) as the chromogen, resulting in a brown cytoplasmic staining in MPO-positive inflammatory cells. Nuclear counterstaining was performed using Harris’ hematoxylin for 2 min. Finally, sections were dehydrated, cleared in xylene, and mounted using DPX. MPO expression was semi-quantitatively evaluated based on the percentage and intensity of positively stained inflammatory cells in the alveolar septa and perivascular areas. Histological and immunohistochemical evaluations were performed using an Olympus CX40 light microscope Olympus Optical Co., Ltd., Shinjuku, Tokyo, Japan).

### 4.17. Statistical Analysis

Data were statistically analyzed using one-way analysis of variance (ANOVA), followed by Tukey’s post hoc multiple comparison test, using SPSS (version 27; IBM Corp., Armonk, New York, USA). All data were tested for normality and homogeneity of variances before analysis. Results are expressed as mean ± standard deviation (SD). Statistical differences among groups were considered significant at *p* < 0.05.

## 5. Conclusions

In conclusion, taurine-loaded chitosan nanoparticles effectively counteract dexamethasone-induced tissue damage, outperforming free taurine and silymarin in restoring hematological balance, antioxidant defenses, thyroid hormonal function, and pulmonary and thyroid histoarchitecture. The nanoscale formulation enhances bioavailability, promotes targeted tissue delivery, and sustains therapeutic efficacy, underscoring its potential as a promising strategy for protecting against steroid-induced organ injury. These findings provide a solid foundation for future translational studies exploring taurine nanoformulations in clinical or preclinical settings.

## Figures and Tables

**Table 1:** Effect of taurine and taurine-loaded nanoparticles on hematological parameters in dexamethasone-treated rats.

Groups/Parameters	Hgb (g/dL)	Leukocyte (×10^3^/mm^3^)	Neutrophil%	Lymphocyt%	Eosinophil%	Monocyte%
**Control**	13.45 ± 0.34	6.32 ± 0.15	62.34 ± 0.37	34.96 ± 0.22	1.60 ± 0.19	1.63 ± 0.15
**DEXA**	10.49 ± 0.2 ^***^	8.95 ± 0.40 ^***^	67.65 ± 0.40 ^***^	24.96 ± 0.22 ^***^	0.20 ± 0.04 ^***^	0.48 ± 0.05 ^***^
**DEXA + Sily**	12.09 ± 0.13 ^**, #^	7.84 ± 0.31 ^**, #^	64.95 ± 0.19 ^**, #^	29.21 ± 0.27 ^**, #^	0.81 ± 0.07 ^**, #^	1.03 ± 0.04 ^**, #^
**DEXA + Tau**	12.50 ± 0.14 ^*, ##^	7.02 ± 0.11 ^*, ##^	63.45 ± 0.41 ^*, ##^	32.02 ± 0.14 ^*, ##^	1.07 ± 0.03 ^*, ##^	1.30 ± 0.03 ^*, ##^
**DEXA + Tau-NPs**	12.86 ± 0.09 ^NS, ###^	6.68 ± 0.33 ^NS, ###^	62.41 ± 0.38 ^NS, ###^	34.84 ± 0.54 ^NS, ###^	1.39 ± 0.16 ^NS, ###^	1.48 ± 0.06 ^NS, ###^

Data are presented as Mean ± SD (*n* = 8). Statistical analysis was performed using one-way ANOVA followed by Tukey’s post hoc test. *** *p* < 0.001, ** *p* < 0.01, and * *p* < 0.05 indicate significant differences compared with the control group. #, ##, and ### indicate significant differences compared with the dexamethasone (DEXA) group at *p* < 0.05, *p* < 0.01, and *p* < 0.001, respectively. NS: non-significant compared with the control group. Hgb: hemoglobin.

**Table 2:** Effect of taurine and taurine-loaded nanoparticles on oxidative stress and antioxidant markers in lung tissue of dexamethasone-treated rats.

Groups/ Parameters	CAT (U/mg Protein)	MDA nmol/g Tissue	SOD U/mg Protein)	GSH mg/g Tissue
**Control**	7.30 ± 0.33	110.93 ± 1.63	5.22 ± 0.19	13.32 ± 0.25
**DEXA**	4.06 ± 0.07 ^***^	173.83 ± 8.20 ^***^	2.48 ± 0.03 ^***^	7.13 ± 0.36 ^***^
**DEXA + Sily**	5.09 ± 0.12 ^**, #^	141.33 ± 3.07 ^**, #^	3.42 ± 0.03 ^**, #^	10.15 ± 0.17 ^**, #^
**DEXA + Tau**	6.12 ± 0.11 ^*, ##^	125.00 ± 3.03 ^**, ##^	4.03 ± 0.06 ^*, ##^	11.05 ± 0.20 ^*, ##^
**DEXA + Tau-NPs**	7.14 ± 0.09 ^NS, ###^	114.83 ± 1.94 ^*, ###^	5.09 ± 0.12 ^NS, ###^	13.68 ± 0.24 ^NS, ###^

Data are expressed as Mean ± SD (*n* = 8). Statistical comparisons were performed using one-way ANOVA followed by Tukey’s post hoc test. *** *p* < 0.001, ** *p* < 0.01, and * *p* < 0.05 indicate significant differences compared with the control group. #, ##, and ### indicate significant differences compared with the dexamethasone (DEXA) group at *p* < 0.05, *p* < 0.01, and *p* < 0.001, respectively. NS: non-significant compared with the control group. CAT: catalase; MDA: malondialdehyde; SOD: superoxide dismutase; GSH: reduced glutathione.

**Table 3:** Effect of taurine and taurine-loaded nanoparticles on thyroid oxidative stress, inflammation, and detoxification markers in dexamethasone-treated rats.

Groups/ Parameters	MDA (nmol/g Tissue)	SOD (U/mg Protein)	GSH (mg/g Tissue)	NO (nmol/g Tissue)	QR (Pg/g Tissue)	GGT (U/L)
**Control**	5.63 ± 0.14	22.59 ± 0.34	24.68 ± 0.26	7.64 ± 0.04	114.29 ± 0.63	29.51 ± 0.24
**DEXA**	23.42 ± 0.34 ^***^	15.11 ± 0.14 ^***^	5.76 ± 0.25 ^***^	28.09 ± 0.05 ^***^	39.81 ± 0.02 ^***^	106.28 ± 0.57 ^***^
**DEXA + Sily**	15.34 ± 0.39 ^***, #^	16.06 ± 0.08 ^**, #^	8.53 ± 0.19 ^***, #^	19.21 ± 0.25 ^***, #^	52.08 ± 0.13 ^***, #^	68.33 ± 0.26 ^***, ##^
**DEXA + TAU**	11.41 ± 0.24 ^**, ##^	17.46 ± 0.29 ^**, ##^	19.15 ± 0.21 ^*, ##^	13.53 ± 0.51 ^**, ##^	69.26 ± 0.35 ^**, ##^	45.19 ± 0.27 ^**, ##^
**DEXA + Tau-NPs**	9.12 ± 0.09 ^*, ###^	19.19 ± 0.18 ^*, ###^	23.60 ± 0.28 ^NS, ###^	11.00 ± 0.19 ^*, ###^	78.41 ± 0.29 ^**, ###^	38.09 ± 0.12 ^*, ###^

Data are expressed as Mean ± SD (*n* = 8). Statistical analysis was carried out using one-way ANOVA followed by Tukey’s multiple comparison test. *** *p* < 0.001, ** *p* < 0.01, and * *p* < 0.05 indicate significant differences compared with the control group. #, ##, and ### indicate significant differences compared with the dexamethasone (DEXA) group at *p* < 0.05, *p* < 0.01, and *p* < 0.001, respectively. NS: non-significant compared with the control group. MDA: malondialdehyde; SOD: superoxide dismutase; GSH: reduced glutathione; NO: nitric oxide; QR: quinone reductase; GGT: gamma-glutamyl transferase.

## Data Availability

All data generated or analyzed during this study are included in this published article.

## Abbreviations

Tau–CS NPs	Taurine-loaded chitosan nanoparticles
Tau-NPs	Taurine nanoparticles
DEXA	Dexamethasone
Hgb	Hemoglobin
CAT	Catalase
SOD	Superoxide dismutase
GSH	Reduced glutathione
MDA	Malondialdehyde
NO	Nitric oxide
QR	Quinone reductase
GGT	Gamma-glutamyl transferase
T3	Triiodothyronine
T4	Thyroxine
TSH	Thyroid-stimulating hormone
MPO	Myeloperoxidase
H&E	Hematoxylin and eosin
UV–vis	Ultraviolet–visible spectroscopy
FTIR	Fourier-transform infrared spectroscopy
EE%	Encapsulation efficiency
DLC%	Drug loading capacity
